# Effects of Polystyrene Diet on the Growth and Development of *Tenebrio molitor*

**DOI:** 10.3390/toxics10100608

**Published:** 2022-10-13

**Authors:** Xiaosu Wang, Tianle Tang

**Affiliations:** 1 School of Tropical Medicine, Hainan Medical University, Haikou 571199, China; 2 NHC Key Laboratory of Tropical Disease Control, Hainan Medical University, Haikou 571199, China

**Keywords:** *Tenebrio molitor*, polystyrene (PS) foam, larval growth, electron microscopy, omics technology

## Abstract

In recent years, the role of *Tenebrio molitor* in degrading polystyrene foam through its gut microbes has become the focus of research. However, little literature has reported the effect of feeding on polystyrene foam on the growth and development of *Tenebrio molitor*. In this study, we investigated the impacts of different polystyrene by evaluating the vital signs of *Tenebrio molitor* fed in the intestines and excrement fluids using RNA-Seq t.echnology and then verifying the transcriptome sequencing findings using qRT-PCR technology. The average weight of *Tenebrio molitor* larvae in the wheat bran group increased significantly. *Tenebrio molitor* larvae in the PS group, on the other hand, didn’t grow as much and had a much lower average weight than those in the wheat bran group. Compared to the bran group, the excrement of *Tenebrio molitor* fed only on polystyrene foam was flaky and coarse, increased nitrogen and phosphorus atomic concentration ratios by about 50%, decreased potassium atomic concentration ratios by 63%, with the enterocytes and circular muscle of *Tenebrio molitor* falling as well. Kyoto Encyclopedia of Genes and Genomes enrichment indicated that the differential genes were mainly related to metabolic pathways. There was an agreement between qRT-PCR and RNA-Seq analyses for the growth and development genes chitinase, heat shock protein 70, and cytochrome P450. Only feeding polystyrene foam shall lead to the growth and development retardation of *Tenebrio molitor*.

## 1. Introduction

Plastics are now ubiquitous in all facets of human existence and the global economy. However, the widespread use of plastics and the uncontrolled dumping of plastic waste result in plastic pollution. The great majority of plastic waste comes from the packaging industry, where one of the main types of resins used in production is polystyrene (PS) which accounts for 7% of all plastics. PS is a synthetic aromatic polymer made from styrene monomer. PS causes not only environmental pollution but also human health problems and ecosystem changes due to its toxicity [1]. PS has long-term persistence and strong resistance to degradation [2]. Once PS enters the environment, it will exist for a long time and cause environmental pollution. If current production and waste management trends continue, approximately 12 billion tons of plastic waste will accumulate in the natural environment and landfills by 2050 [3,4], which will have potentially severe consequences for the oceans and wildlife, with incalculable long-term effects on the ecosystems. In recent years, the discovery of insect degradation foam such as *Tenebrio molitor* (*T. molitor)*, *Galleria mellonella*, *Zophobas atratus Larvae*, and *Bombyx mori* [5,6,7,8,9,10,11], and the research of intestinal functional microorganisms [12,13,14,15], provides a new research direction for efficient biodegradation of waste plastics.

*T. molitor*, also known as mealworm, belongs to the Coleoptera, Tenebrionidae. It has a life cycle of 90–100 days [16]. *T. molitor* has good nutrition, low feeding costs, a protein content of 49–55%, an amino acid type and proportion close to the standard recommended by WHO, a high unsaturated fatty acid content, and a lot of chitosan. It is an insect resource with great development potential [17,18,19]. Studies have shown that using *T. molitor* powder instead of conventional protein feed has no significant adverse effects on the growth performance of animals or the quality of livestock products [20,21,22]. In 2021, the European Commission issued Regulation (EU) No. 2021/8820 approving dried *T. molitor* larvae for marketing as a new resource food, which is the first new resource food approved for insects in the EU [23]. Although *T. molitor* powder is a new food raw material certified by the European Union, its safety should be paid attention to when applied to food [24,25,26]. During the larval stage, mealworms feed on wheat bran, vegetation, and dead insects. *T. molitor* is one of the few insect species that can break down lignocellulosic substrates of resistant cellulosic wastes (like cardboard) and plastic wastes (like polystyrene, polyethylene, polypropylene, and polyvinyl chloride) [27]. Compounds produced during the degradation of polystyrene by *T. molitor* larvae include the trimers 2,4,6-triphenyl-1-hexene and 1,3,5-triphenylcyclohexane [28], the volatiles acetophenone and cumyl alcohol, and 2,4-di-tert-butylphenol, a non-intentionally added substance (NIAS) present in the plastic material [29]. The extract also contains PS monomer styrene and α-methyl styrene. It is reported that 35% to 50% of the ingested polymer is converted into CO_2_ [6,15,30]. Based on the fact that *T. molitor* can break down plastic, adding PS foam to its diet can speed up the rate at which plastic breaks down.

Polystyrene foam is an irreplaceable material in many fields, so how to deal with plastic waste has become an increasingly severe environmental problem. Under the influence of the concept of sustainable development, the characteristic of *T. molitor* of degrading polystyrene foam has received a great deal of research attention, with the role of gut microbes in degrading foam plastics being the focus. However, little literature has reported the effects of feeding PS foam on the growth and development of *T. molitor*. This study analyzes and evaluates the vital signs, intestinal histology, and gene expression changes of *T. molitor* under different feed ratios to provide a scientific reference for the feasibility of feeding polystyrene foam to *T. molitor*.

## 2. Materials and Methods

### 2.1. Animals and Treatments

*T. molitor* larvae and wheat bran were purchased from Tian Tai Insect Breeding Corporation, Sichuan, China. The larvae were identified based on morphology, and before testing, wheat bran was used as their source of nutrition for 7 days. *T. molitor* larvae (developmental stage: instar 5–7) used in this study were cultured at Hainan Medical University (Haikou, China), and the larvae were kept in the breeding room at 26 ± 1 °C with a relative humidity of 65% ± 5%.

Three hundred larvae were divided into 2 groups (polystyrene foam group and wheat bran control group). An amount of 150 larvae were randomly selected group and fed with 1.0 g polystyrene foam blocks (1.5–2.0 cm thickness, purchased from the local packaging market, Haikou, China) and wheat bran as a sole diet, respectively. Subsequently, *T. molitor* larvae were transferred to a clean box to collect the excrement every 12 h and avoid carryover of un-ingested Styrofoam morsels mixing with the accumulated feces. The color of its *T. molitor* excrement is brown, and the color of polystyrene foams is white. The collected excrement was immediately stored for further analysis. *T. molitor* larvae were counted and weighed every ten days. The dead were removed, and the residual PS was weighed. This process continued until the fortieth day.

### 2.2. Physical Representation of Excrement and Histology Observation of Intestinal Tract

The physical and chemical properties of yellow mealworms’ excrement after eating plastic alone and wheat bran control were tested by Phenom ProX scanning electron microscope (SEM, Thermo Fisher Scientific, Massachusetts, USA) and a specially designed and fully integrated apparatus, Energy Dispersive Spectrometer (EDS). Tests were carried out in the State Key Laboratory of Marine Resources Utilization in the South China Sea, Hainan University, China. Intestines stored in 10% buffered formalin were embedded in paraffin, cut at 5 μm, and stained with hematoxylin and eosin. These sections were then examined for histoarchitectural changes under an optical microscope (E200 MV, Nikon, Tokyo, Japan).

### 2.3. Illumina Sequencing for Transcriptome Analysis

*T. molitor* larvae from the treated and control groups were added to 1 mL of Trizol reagent (Invitrogen, Carlsbad, CA, USA) to extract the total RNA. RNA purity and integrity were checked with a NanoDrop spectrophotometer (IMPLEN, Westlake Village, CA, USA). RNA-Seq was conducted by Sagene Biotech Co., Ltd. (Guangzhou, China) and HiseqPE150 (Illumina, Inc., San Diego, CA, USA). Based on the sequence of Tribolium castaneum as the reference genome, differential expression analysis of the samples with three biological replicates was performed using the edgeR package (3.30.0) in a threshold criterion of the value of |log2FC| > 1 and FDR < 0.05. The annotations of differentially expressed genes (DEGs) were based on the Gene Ontology (GO) and Kyoto Encyclopaedia of Genes and Genomes (KEGG) databases.

### 2.4. Quantitative Real-Time PCR (qRT-PCR) Validation

After treatment for 40 days, polystyrene foam groups and wheat bran control groups were prepared to perform the qRT-PCR study. The purpose of this study was to confirm the accuracy of RNA-seq results. 3μg total RNA of each group was reverse-transcribed using the GoScript Reverse Transcription System (A5001, Promega, Madison, WI, USA) according to the manufacturer’s instructions. The qRT-PCR amplifications were performed with the TIAN LONG TL988-IV (Xi’an, China), and GoTaq qPCR Master Mix (A6002, Promega, Madison, WI, USA) was applied to conduct three biological replications and three technical replications to all genes in each pool. The reaction profile was as follows: initial denaturation at 95 °C for 10 min, followed by 40 cycles of denaturation at 95 °C for 15 s, and annealing/elongation at 60 °C for 60 s. RpS3 served as the housekeeping gene. qRT-PCR was performed on Unigene028165, Unigene040524 and Unigene049660 to observe expression level (Table 1). The relative mRNA expression levels of antioxidant response-related genes were analyzed with the method of 2^−ΔΔCt^. This study employed GraphPad Prism 9.0.0 computer program (GraphPad Software, Inc., San Diego, CA, USA) to conduct statistical analysis and the rest of the data were presented as mean ± standard error of the mean (SEM). One-way analysis of variance (ANOVA) and Dunnett’s test were used to evaluate the differences between groups (* *p* < 0.05; ** *p* < 0.01).

## 3. Results and Analysis

### 3.1. Vital Signs of Tenebrio molitor Larvae and Degradation Rate of PS

**Figure 1:**
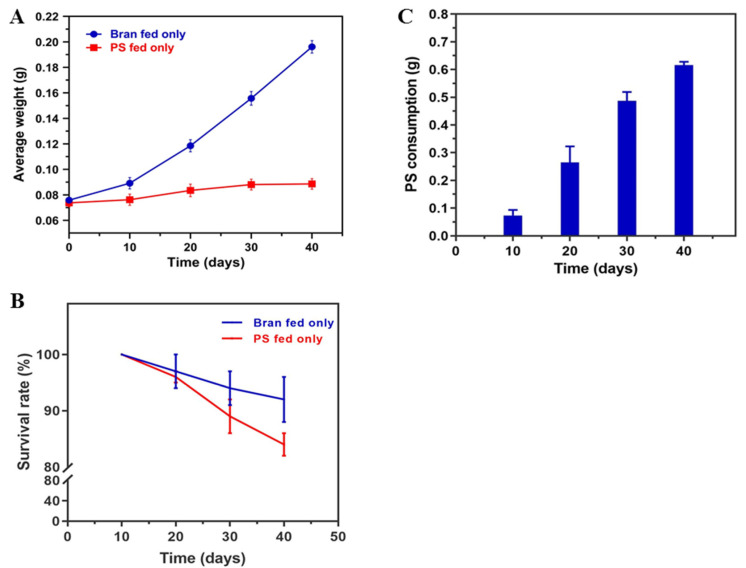
Vital signs of *T. molitor* larvae and degradation rate of PS. (**A**). The blue and red curves represent the average weight of the control group (wheat bran group) and the PS group (PS foam only), respectively. (**B**). Survival rates of *T. molitor* fed PS only compared with that fed bran. (**C**). The blue bar chart represents the cumulative consumption of PS by mealworms (measured every 10 days). The results are presented as mean ± standard error of mean (SEM) (fifty larvae as a group, n = 3).

Throughout the experimental period, the average weight of *T. molitor* larvae in the wheat bran group increased remarkably. Wheat bran can provide nutrients such as crude protein, amino acids, fats, and fatty acids for *T. molitor* [31]. In contrast, the average weight of *T. molitor* larvae in the PS group had limited growth and was substantially lower than that in the wheat bran group (Figure 1A). From 0 (instar 5–7) to 40 days, the survival rate of *T. molitor* in the PS group was lower than that in the wheat bran group (Figure 1B). We found that mealworms continued to consume PS throughout the entire 40-day experiment but at a faster rate over the observation range of 10 to 30 days (Figure 1C).

### 3.2. Physical and Chemical Representation of Excrement

**Figure 2:**
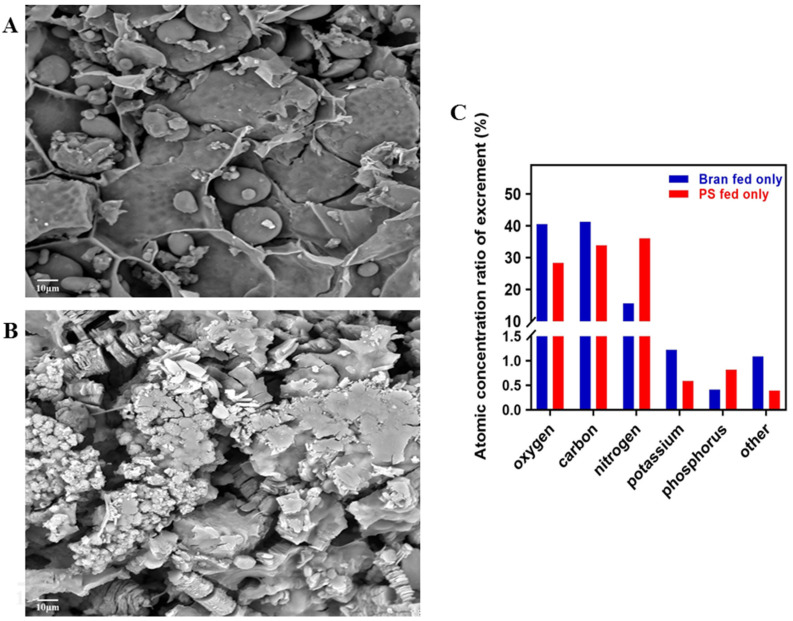
Physical and chemical representation of excrement. (**A**). The excrement of bran-fed larvae. (**B**). The excrement of PS-fed larvae. (**C**). The ratio of atomic concentrations of major elements in excrement in the bran and PS groups.

After 40 days of feeding, the excrement of larvae fed on wheat bran was spherical and smooth (with an average diameter of 15–20 nm) (Figure 2A). As shown in Figure 2B, the excrement of larvae fed PS was flaky and coarse (with an average diameter of 10 nm). The chemical element composition of the excrement was obtained by a fully integrated energy dispersive spectrometer (EDS). In the wheat bran group, the atomic concentration ratios of the composition of the main elements were 40.45%, 41.25%, 15.58%, 1.22%,0.41%, and 1.09% were associated with oxygen (O), carbon (C), nitrogen (N), potassium (K), phosphorus (P), and other chemical elements, respectively. In contrast, the PS group, these ratios were 28.31%, 33.83%, 36.06%, 0.59%, 0.82%, and 0.39% for O, C, N, K, P, other chemical elements, respectively (Figure 2C). It was clear that the ratio of atomic concentrations of N and P in the excrement of the PS group was about 50% higher than that of the bran group, and that the ratio of atomic concentrations of K was 63% lower than that of the bran group.

### 3.3. Midgut of Tenebrio molitor Larva

**Figure 3:**
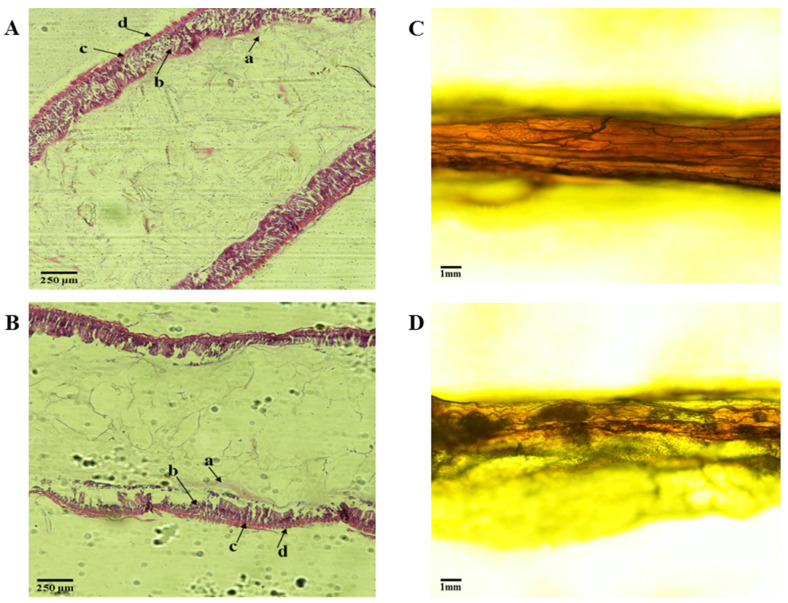
Paraffin tissue in the midgut of *T. molitor* larva (HE staining), including mealworms fed with wheat bran only (**A**) and mealworms fed with PS only (**B**). a: peritrophic membrane; b: enterocytes; c: circular muscle; d: longitudinal muscle. (**C**). Midgut morphology of *T. molitor* larvae fed wheat bran. (**D**). Midgut morphology of *T. molitor* larvae fed with PS.

As indicated in Figure 3A,B, mealworms are relatively symmetrically in the midgut, with a thin muscle layer and a unique nutritional membrane (a). Compared with the wheat bran-fed control group, the enterocytes (b) and circular muscle (c) of the PS-fed group were decreased, especially the enterocytes associated with digestion. The midgut of *T. molitor* in the wheat bran group is full and round (Figure 3C), while that in the PS group is rough (Figure 3D).

### 3.4. Transcriptome Analysis and qRT-PCR Verification

#### 3.4.1. Statistics of Transcriptome Sequencing Yield

Using the Illumina sequencing platform to sequence *T. molitor* transcriptome, the filtered reads of each sample are between 51 million and 66 million, and the filtered reads ratio is above 99.70%. The filtered GC content is between 45.60% and 47.63%, with Q20 (percentage of phred quality score > 20) values greater than 97%, and Q30 (percentage of phred quality score > 30) values greater than 93%. The results indicated that the quality of the samples and data were high, and subsequent experiments and analyses could be conducted.

#### 3.4.2. Gene Ontology (GO) Functional Classification of Transcriptome

There were 111,233 unigenes in the transcriptome process. GO functional annotation can be obtained according to Nr annotation information. GO is an internationally standardized gene function classification system that provides dynamically updated standards.

**Figure 4:**
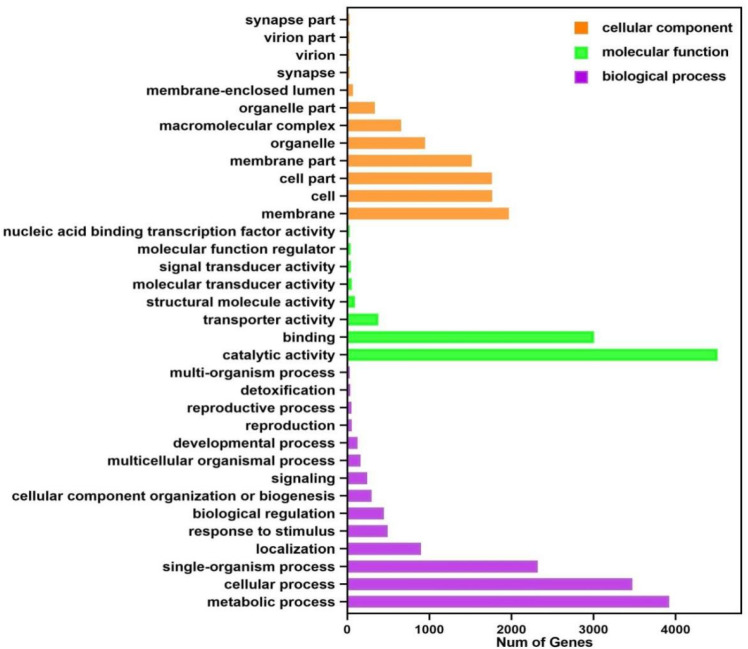
GO Function Classification.

Annotations were made for transcripts in three GO functional categories: molecular function, biological process, and cellular component. As illustrated in Figure 4, among the biological process categories, metabolic (3920 transcripts), cellular (2322 transcripts), and single organisms (4429 transcripts) were the most annotated small functional categories. Among the cellular component categories, the most annotated were membrane (1968 transcripts), membrane component (1515 transcripts), cell (1764 transcripts), and cellular component (1762 transcripts). While among the molecular function categories, catalytic action (4511 transcripts) and binding (3007 transcripts) were the most annotated small functional categories.

#### 3.4.3. KEGG Enrichment Analysis of DEGs

**Figure 5:**
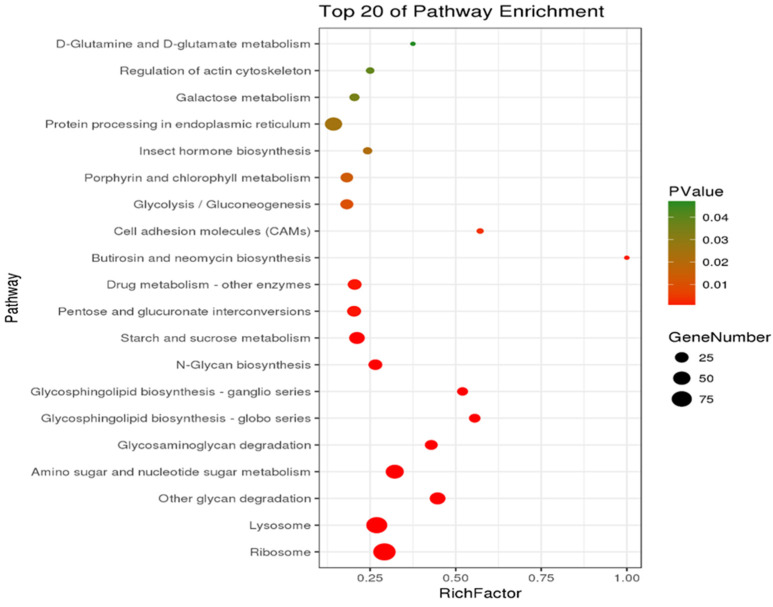
Diagram of CK-vs-PS KEGG enrichment analysis.

KEGG enrichment analysis was performed on the samples with a significant enrichment criterion *p*-value < 0.05 (*p*-value after correction for multiple hypothesis testing). Results were presented in Figure 5, with the top pathways of the Rich Factor sorted in descending order.

There were a total of 733 DEGs in the PS foam group and the wheat bran control group. These DEGs were enriched in 178 KEGG pathways distributed in five major categories, i.e., metabolism (75 pathways), organismal systems (51 pathways), environmental information processing (19 pathways), genetic information processing (20 pathways), and cellular processes (13 pathways). As shown in the scatterplots, there were 4 significantly enriched pathways. Butirosin and neomycin biosynthesis (3 DEGs, 0.41%), Cell adhesion molecules (4 DEGs, 0.55%), Glycosphingolipid biosynthesis–globo series (15 DEGs, 2.05%), Glycosphingolipid biosynthesis-ganglion series (13 DEGs, 1.77%). This indicated that the metabolic process of the intestinal tract of *Tenebrio* larvae fed on PS on a long-term basis was greatly affected. The significantly enriched cell adhesion molecule pathway belonged to the category of environmental information processing, and the remaining three significantly enriched pathways belonged to the metabolism category. It is speculated from the pathway functions that these pathways may be related to *T. molitor* metabolism.

#### 3.4.4. Genetic Analysis and qRT-PCR Verification

**Figure 6:**
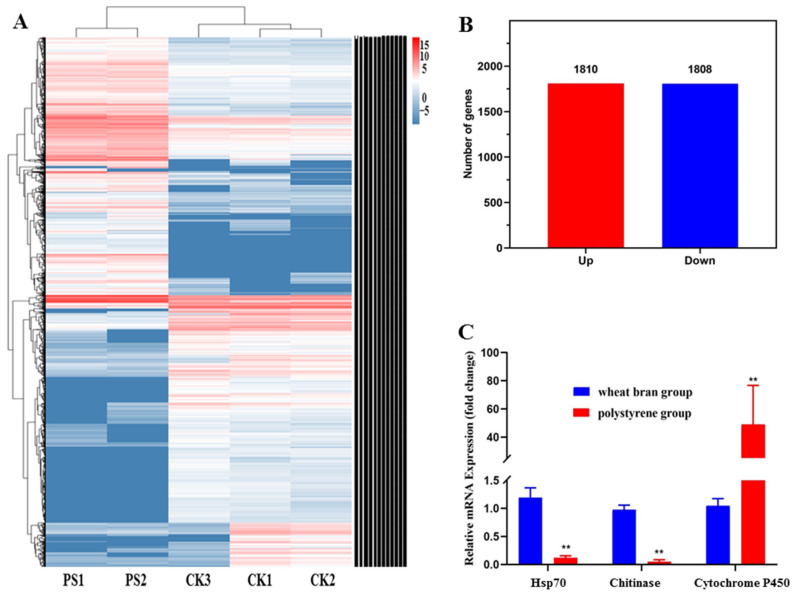
A. Clustering diagram of differential gene expression patterns among groups. B. Differential expression gene statistics between groups. C. qRT-PCR validation of DEGs. One-way analysis of variance (ANOVA) and Dunnett’s test were used to evaluate the differences between groups (** *p* < 0.01).

Normally, a gene is considered differentially expressed if it is more than two-fold differentially expressed in two groups of samples. Differences in gene expression will be significant with the FDR value decrease. We used FDR and log2FC to screen for differential genes, and the screening conditions were FDR < 0.05 and |log2FC| > 1. As indicated in Figure 6B, 1810 DEGs were up-regulated, while 1808 DEGs were down-regulated among the screened DEGs.

We calculated the logarithm of the gene expression level of each sample with base 2, and performed hierarchical cluster analysis on different samples and genes based on the gene expression. Heat maps were used to present the clustering results, with each column and row representing a sample and a gene, respectively. The expression level of genes in different samples was shown in different colors, with redder colors indicating higher expression while bluer colors represented the opposite.

From the sample clustering results in Figure 6A, it was found that the five samples were divided into two categories: PS1 and PS2 were in one category, while CK1, CK2, and CK3 were clustered in another. By comparing the expression levels of each gene in different groups, we found that *T. molitor* larvae had significant differences in intestinal gene expression levels when feeding on PS and wheat bran. This intended that PS consumption had a more significant effect on the intestinal tract of *T. molitor* larvae.

Three genes related to growth and development, Hsp70 (Unigene028165), chitinase (Unigene040524), and cytochrome P450 (Unigene049660), were selected for the qRT-PCR test. The results showed that the expression trends of the three genes in the PS foam group and the wheat bran control group were consistent in the transcriptome and qRT-PCR, indicating that the transcriptome sequencing results were consistent (as shown in Figure 6C).

## 4. Discussion

### 4.1. Vital Signs of Tenebrio molitor Larvae and Degradation Rate of PS

Owing to the marked impact of environmental factors, such as temperature and humidity, upon the growth and reproduction of *T. molitor* [32], these two factors were precisely controlled during the test, reducing the interference of irrelevant variables. After the experiment, the average weight and survival rate of *T. molitor* and the loss of PS foam were measured. According to the experimental data, the average weight of *T. molitor* larvae in the wheat bran group increased significantly. In contrast, the average weight of *T. molitor* larvae in the PS group had limited growth and was substantially lower than their wheat bran counterparts. *T. molitor* was unable to utilize PS as its only source of C. The energy released through PS digestion does not account for the energy required for nourishment [33,34]. From 0 (instar 5–7) to 40 days, the survival rate of *T. molitor* in the PS group was lower than that in the wheat bran group. This study also found that *T. molitor* could continue to consume PS throughout the 40-day experiment but at a faster rate during 10–30 days. This suggested that PS foam did not negatively affect the viability of *T. molitor*, which was consistent with the study of Yong Yang and Qingqing Wu [12,35].

### 4.2. Physical and Chemical Characterization of Tenebrio molitor Excrement

It is worth noting that *T. molitor* also produced a large amount of excrement while degrading PS foam. The experimental results showed that the excrement of the bran-fed larvae was spherical and smooth, while those fed only on PS were flaky and coarse. Research has found that the excrement of *T. molitor* contains plenty of organic matter and nutrients such as N, P, and K, and it has become a critical biomass raw material. In this study, the K atomic concentration ratio in the excrement of *T. molitor* fed only PS was 63% lower than in the bran group. Low K concentrations were so harmful to insects that K is commonly used as a supplement for insect nutrition [36,37]. Consequently, *T. molitor* may also require high K levels for normal development. The ratio of N to P atoms in the excrement of the PS group increased by about 50% compared to the bran group, which demonstrated that eating PS particles had a particular effect on the growth of *T. molitor*. This result may be related to the inability of *T. molitor* larvae to obtain sufficient energy from food and thus consume their lipids. The most significant changes were recorded in the levels of triglycerides, which is in line with the fact that oxidation of fatty acids and glycerol (stored as tri-glycerides) is the essential source of energy for insects. Fat is accumulated in the insect fat body [38]. It may also be related to the activity of the intestinal microorganisms of *T. molitor*. Interestingly, according to previously published reports, the gut microbiome of *T. molitor-fed* PS were Enterobacter, Klebsiella, Streptococcus, Lactococcus, Enterococcus, etc. [39,40,41]. The degradation of PS may be attributed to the direct biochemical action of intestinal bacteria or in combination with the larval digestive system. *T. obscurus* chews up the plastic foam into small pieces. This increases the polymer’s surface area and makes it easier for extracellular enzymes to break it down [1,42]. The effective biodegradation of PS is achieved through the synergistic effect of the larvae of *T. molitor* and their intestinal microbial activity. Further studies are recommended to investigate the synergistic effect between *T. molitor* digestion and microbial metabolism and better understand the enzymatic systems involved in the biodegradation of PS plastics.

### 4.3. Histological Observation on Intestinal Tract of Tenebrio molitor

The PS-fed group had fewer enterocytes and circular muscles than the wheat bran-fed group, which was a significant difference that could be seen under a microscope investigation. The midgut morphology of *T. molitor* in the bran group was round, and the morphology of the PS group was rough, so feeding only PS affected the intestinal digestion of *T. molitor*. This may be one of the reasons why feeding PS foam alone leads to retarded growth and development of *T. molitor*. The exact mechanism of how PS foam affects the reduction of intestinal enterocytes and circular muscles of *T. molitor* remains to be further studied.

### 4.4. Transcriptomic Analysis and qRT-PCR Test of Tenebrio molitor

The results of the KEGG pathway enrichment analysis revealed that the metabolic processes in the intestine of *T. molitor* larvae fed on PS for a long time were greatly affected. The significantly enriched cell adhesion molecule pathway belonged to the category of environmental information processing, and the remaining three significantly enriched pathways belonged to the metabolism category. Based on how the pathways work, it is thought that these pathways may be related to the metabolism of *T. molitor*. The reduction in food intake leads to starvation of *T. molitor*, affecting energy metabolism. The trehalose level is closely related to metabolic homeostasis [43]. Plastic exposure inhibited the expression levels of trehalose transporter (TRET1) and trehalose 6-phosphate synthase/phosphatase (TPS), the regulatory genes involved in trehalose transport and metabolism. In particular, the expression of TPS dropped by 50%, which shows that the amount of trehalose in insects dropped after they had been exposed to plastic for a long time [44]. Trehalose is a vital sugar source widely existing in organisms, which can protect organisms from environmental stress [45]. Interestingly, trehalose metabolism genes influence chitinase metabolism, particularly chitinase expression [46]. The results of qRT-PCR showed that chitinase, cytochrome p450, and heat shock protein 70 had certain effects on the development of *T. molitor*.

*T. molitor* molts regularly shed their exoskeletons and synthesize new ones to maintain normal growth and development. This is the chitin metabolism process of *T. molitor*, which is completed by the synergistic action of chitin synthesis and enzymatic degradation. Insect chitinase is a kind of hydrolase that can inhibit or degrade chitin, including endochitinase and exochitinase [47]. It primarily involves essential physiological processes such as exuviation, peritrophic membrane degradation, cell proliferation, and immune defenses. It affects the growth of insects by regulating the degradation of chitin [48,49]. The failure of chitinase 5 to be appropriately expressed causes loss of larval-larval and pupal-adult development, indicating that the formation of the new epidermis and the breakdown of the old epidermis cannot be achieved without chitinase 5 [50,51]. Silencing chitinase 5 can prevent completely metamorphosed insects from breaking through the pupal shell in the pupal stage, resulting in the failure of Insect eclosion. Intake of PS can disrupt the normal regulation of chitinase and then affect the development of *T. molitor* by disrupting the chitin metabolism process. The mechanisms by which different types of chitinase affect molting development differ, and this needs to be studied in more depth.

Cytochrome P450 is a heme-containing protein with monooxygenase activity, which exists widely in various organisms [52]. It plays an essential role in the growth, development, and feeding of insects, as well as participates in the synthesis and metabolism of endogenous compounds, such as juvenile hormones, ecdysone, and fatty acids in insects [53]. Studies have shown that the high expression or induced expression of the cytochrome P450 gene leads to an increase in enzymes or changes in enzymatic structure. It is directly related to the detoxification ability of insects and can affect the adaptability of insects to the environment [54]. Therefore, while eating PS, *T. molitor* can enhance its immunity by expressing a large amount of cytochrome P450.

Heat shock protein 70 (HSP70) is typically considered a molecular chaperone, which helps nascent peptide chains to fold correctly and facilitates protein transport between cells. Heat stress can increase the activity of superoxide dismutase, thereby protecting cells from damage. HSP70, as a molecular chaperone or activator, participates in heat-stock response (HSR), thus improving the tolerance of the organism. Research findings also suggest that HSP70 is of great importance to the physiological functions of organisms (e.g., regulating insect growth and development), various resistances, and environmental monitoring [55,56].

The qRT-PCR results revealed that the growth and development-related genes of chitinase, cytochrome p450, and HSP70 have essential roles in molting and development in *T. molitor*. During feeding on PS, the growth of *T. molitor* was influenced by disrupting the routine regulation of chitinase, cytochrome P450, and HSP70.

## 5. Conclusions

The qRT-PCR validation demonstrated the expression characteristics of chitinase, cytochrome P450, and HSP70 genes, indicating that these genes are associated with the growth and development of *T. molitor*. This study found that PS foam can provide certain nutrition with *T. molitor*. However, it should be noticed as well that feeding PS foam solely can lead to retarded growth and development of *T. molitor*. Adding wheat bran or alternately feeding PS foam and wheat bran can not only improve the ability of *T. molitor* to degrade PS foam, but also reduce the adverse impact on the development of *T. molitor* mentioned above. Therefore, further research is needed to verify the feasibility of exploiting *T. molitor* to degrade plastics. It is believed that this problem will be solved in the near future, and the method of using *T. molitor* larvae to degrade plastics will also be widely adopted.

## Figures and Tables

**Table 1:** Primers used for the quantification of mRNA expression by qRT-PCR.

Unigene Number	Primers	Protein (& Gene) Description
N/A	F 5′-GTGGTCGTTTCTGGCAAACT-3′ R 5′-CAACACTCCTTGCCTCAACA-3′	Ribosomal protein S3(RpS3)
Unigene028165	F 5′-CCTGGGCACGACCTACTC-3′ R 5′-GGGTGGTTCTGTTACCTTGG-3′	Heat shock protein 70 (YHSP70)
Unigene040524	F 5′-TGGGATGGGTTCTTGCTG-3′ R 5′-GACCTGGGTGGTGGGATG-3′	Chitinase (chit5)
Unigene049660	F 5′-CTTTACCTACCCACGGTTCC-3′ R 5′-AGCATTTACGAATACACACGG-3′	Cytochrome P450 monooxygenase CYP4BN28(N/A)

## Data Availability

The data presented in this study are available on request from the corresponding author. The data are not publicly available due to the fact that the data that support the findings of this study are not publicly available, as they are only a part of a bigger data set that shall be used in the writing of other publications. Data are, however, available from the authors upon reasonable request.
